# Autosomal Dominant Obstructive Sleep Apnea Syndrome Due to the New Variant c.980_984dup in COL1A2: A Case Report

**DOI:** 10.7759/cureus.110316

**Published:** 2026-06-05

**Authors:** Josef Finsterer

**Affiliations:** 1 Neurology, Neurology and Neurophysiology Center, Vienna, AUT

**Keywords:** col1a2 variant, cpap, hearing loss, ptosis, sleep apnea syndrome

## Abstract

Obstructive sleep apnea syndrome (OSAS) is a complex disorder characterized by the collapse of the upper airway during sleep. Although a family history often suggests a hereditary predisposition to OSAS, there is little evidence that it is actually a monogenic disorder. To date, no case of a patient with hereditary OSAS due to a pathogenic mutation in the collagen type I alpha-2 chain (COL1A2) gene has been described.

The patient was a 72-year-old woman who had suffered from insomnia since the age of 35. OSAS was suspected since then, but was only confirmed at the age of 67 by polysomnography. The OSAS was treated with nocturnal active positive airway pressure (APAP), which led to an improvement. Her medical history also included joint hypermobility, brachydactyly, multiple nevi, an umbilical hernia, left-sided tinnitus and hearing impairment, hair loss, hip pain, and ptosis. Since her son also developed OSAS at the age of 34, hereditary OSAS was suspected, and exome sequencing was performed, which revealed the novel variant c.980_984dup (p.Ile329Alafs*72) in the COL1A2 gene. Since OSAS is a known complication of connective tissue disorders (CTDs), the index patient’s OSAS was attributed to the COL1A2 mutation.

This case suggests that OSAS may be hereditary due to a variant in the COL1A2 gene and may represent the dominant phenotypic feature. Physicians should be aware that OSAS can be hereditary; therefore, CTD patients with a positive family history of OSAS should undergo genetic testing for variants in genes associated with CTD.

## Introduction

Obstructive sleep apnea syndrome (OSAS) is a complex disorder characterized by upper airway collapse during sleep. This leads to repeated pauses in breathing, fragmented recovery, and intermittent drops in oxygen levels. Clinical signs and symptoms of OSAS include loud snoring, observed pauses in breathing, repeated nocturnal awakenings with shortness of breath or choking episodes, pauses in breathing noticed by a partner, abnormal breathing patterns (rapid breathing followed by shallow breathing with pauses), excessive daytime sleepiness, and early morning headaches [1]. The consequences affect the cardiovascular, respiratory, and neurocognitive systems [1]. OSAS is more common in men than in women [1]. OSAS can occur in association with central sleep apnea syndrome (CSAS) [2]. CSAS is associated with heart failure, atrial fibrillation, brainstem damage following stroke, malignancies, central nervous system infections, amyotrophic lateral sclerosis (ALS), Parkinson's disease, long-term opioid use, high-altitude exposure, kidney failure, hypothyroidism, and neuromuscular disorders [2]. OSAS is associated with obesity, airway constriction (e.g., narrow throat, large tongue, small mandible), enlarged tonsils, use of sedatives, alcohol or tobacco, hypertension, diabetes, acromegaly, Down syndrome, ALS, chronic nasal congestion, and a family history of OSAS [3].

Although family histories suggest that OSAS may be hereditary, there is little evidence that OSAS is actually a monogenic disorder. To date, only associations between variants of certain genes and OSAS have been described, but a clear causal relationship has yet to be confirmed. Potential genes that may play a role in the pathophysiology of OSAS include FTO, MC4R, TNF-α, ACE, APOE, RMST/NEDD1, ANGPT2, PTGER3, LPAR1, GPR83, ARRB1, DRD1, and HTR2A [4]. No association between OSAS and the collagen type I alpha-2 chain (COL1A2) gene has been reported to date.

The COL1A2 gene is located on chromosome 7 [5]. The COL1A2 gene provides the blueprint for the alpha chain of type I collagen, the most important structural protein in connective tissue, bone, skin, and the cornea. COL1A2 mutations generally lead to weak connective tissue and to distinctive connective tissue disorders (CTDs) such as osteogenesis imperfecta (brittle bone disease), atypical Marfan syndrome, and Ehlers-Danlos syndrome (EDS) [6]. Since the larynx and upper airways contain a great deal of connective tissue, it is conceivable that a weakening of this connective tissue during sleep leads to insufficient support of the structures crucial for respiration and to airway collapse, thereby preventing the maintenance of physiological conditions. A patient with OSAS who also has a pathogenic mutation in the COL1A2 gene has not yet been described.

## Case presentation

**Figure 1:**
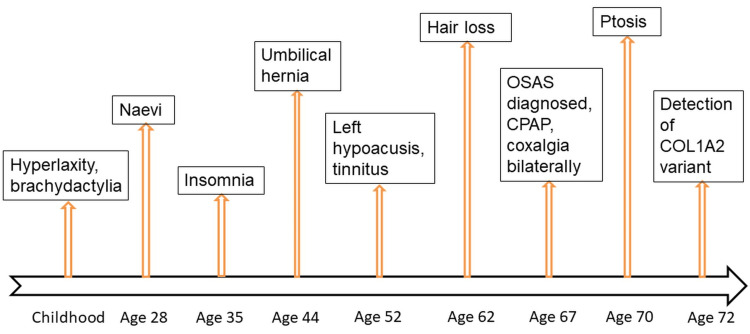
Disease course of the index patient The x-axis shows the patient’s age, and the y-axis shows the onset of phenotypic manifestations.

The patient is a 72-year-old woman (164 cm tall, 64 kg) who has suffered from insomnia since the age of 35. Since then, she has also experienced daytime sleepiness, observed pauses in breathing, morning headaches, and difficulty concentrating. OSAS was therefore suspected but was not confirmed until she was 67. The OSAS was treated with nocturnal active positive airway pressure (APAP). This resulted in improved sleep quality, improved nocturnal oxygenation, and a reduced frequency of breathing pauses. Her medical history also included joint hypermobility and brachydactyly since childhood, multiple nevi beginning at age 28, a hysterectomy at age 44 with complications from an umbilical hernia, tinnitus and hearing impairment in the left ear since age 52, a thyroid nodule detected at age 60, a cholecystectomy at age 61, hair loss since age 62, bilateral coxalgia since age 67, and ptosis since age 70, which required surgical correction at age 71 (Figure 1).

**Figure 2:**
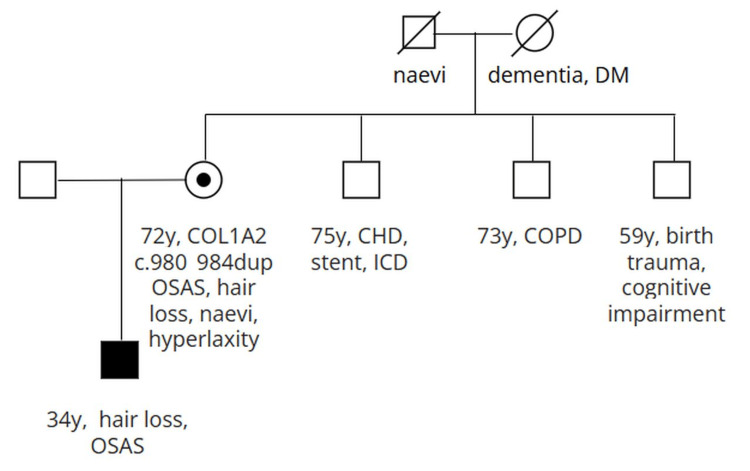
Pedigree of the index patient’s family Only the index patient was genetically tested. The son of the index patient presented with OSAS and hair loss. The father had nevi. All other family members were presumably unaffected. The mutation was not confirmed by Sanger sequencing, and no segregation analysis was performed. CHD: coronary heart disease, COPD: chronic obstructive pulmonary disease, DM: diabetes, ICD: implantable cardioverter defibrillator, OSAS: obstructive sleep apnea syndrome

Her family medical history included the following conditions: OSAS in her son, who was first diagnosed at age 34, hyperlipidemia (son), hair loss (son), dementia and diabetes (mother), coronary artery disease, a stent, and an implantable cardioverter-defibrillator in her 75-year-old brother, chronic obstructive pulmonary disease in her 73-year-old brother, as well as cognitive impairment due to birth trauma in her 59-year-old third brother, and multiple nevi in her father (Figure 2). Due to suspicion of hereditary OSAS, whole-exome sequencing was performed on her at the age of 72, which identified the pathogenic variant c.980_984dup (p.Ile329Alafs*72) in the COL1A2 gene. The patient’s son has so far refused to undergo genetic testing.

**Figure 3:**
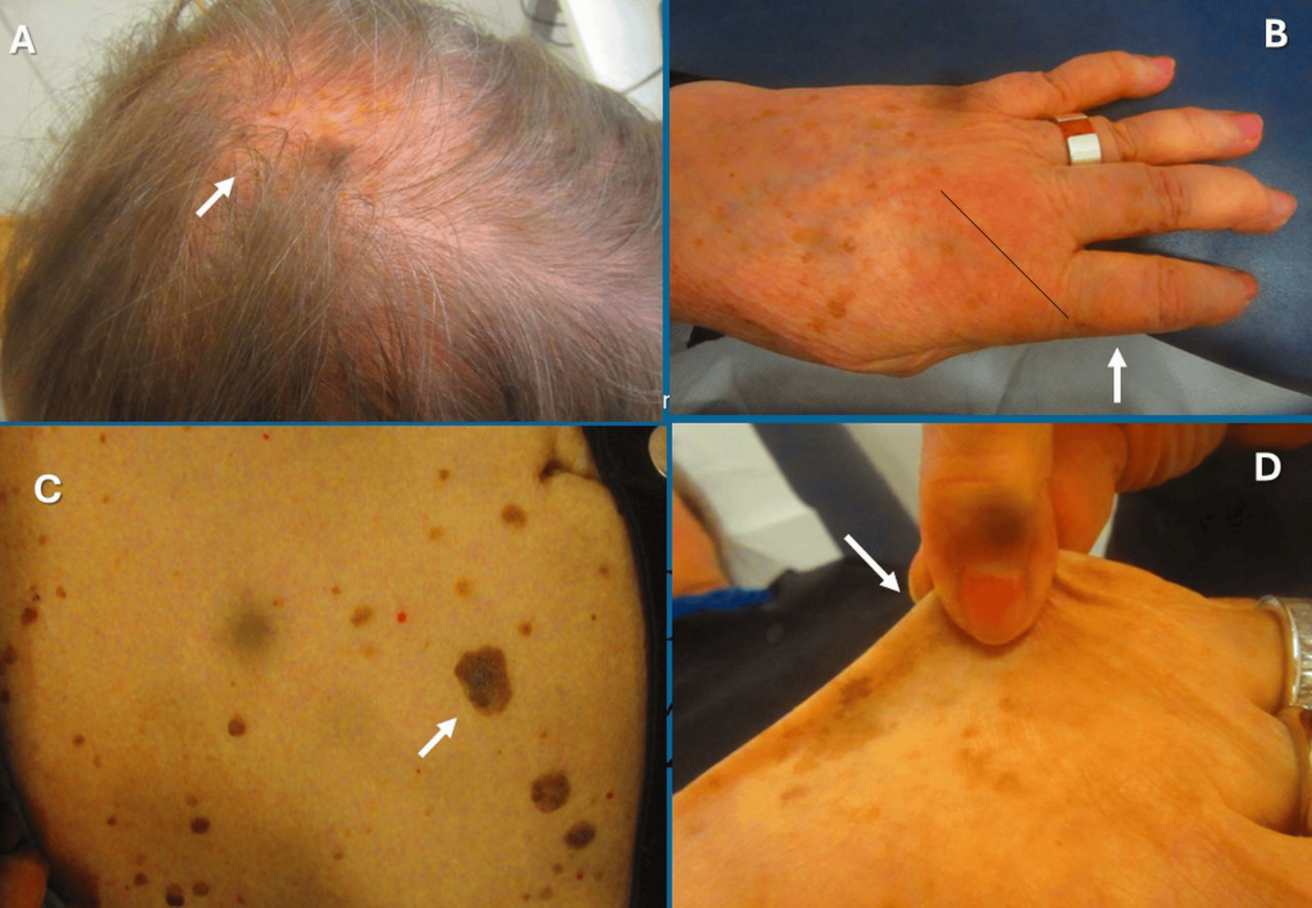
Clinical presentation at age 72 years showing hair loss (panel A), brachydactyly (panel B), multiple nevi (panel C), and hyperlaxity of skin (panel D)

The clinical neurological examination revealed short stature, hair loss, multiple nevi, hypertelorism, myopia, ptosis despite prior surgical ptosis correction, hearing impairment on the left side (for 10 years), brachydactyly, polyarthrosis of the finger joints, hyperlaxity of all joints, hyperlaxity of the skin, paresthesia of the right lower extremity, and flat feet and splayfoot (Figure 3). Blood tests revealed only hyperlipidemia. Nerve conduction studies (NCS) were unremarkable. Brain MRI and EEG were repeatedly unremarkable. Cardiological examinations revealed no ventricular arrhythmias, valvular heart disease, cardiomyopathies, or aortic dilatation or ectasia.

**Figure 4:**
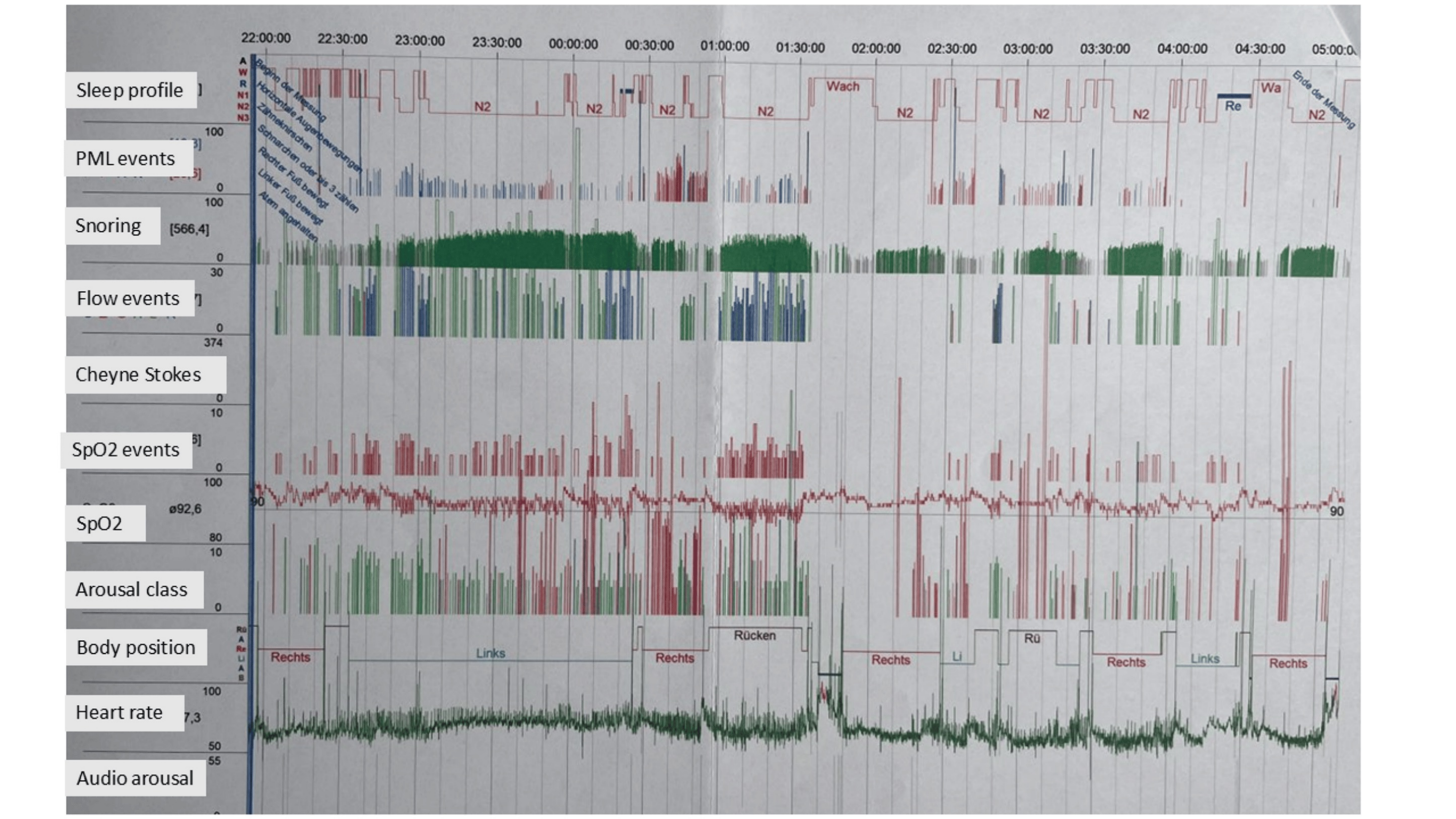
Polysomnography at the age of 71 showing low sleep efficiency with lack of deep sleep and rapid eye movement (REM) phases, frequent arousals, frequent periodic limb movements (PLM), homogenous and intermittent snoring, and an increased apnea/hypopnea index

A polysomnography performed at age 67 revealed a severe disturbance in sleep architecture on the hypnogram, with a marked deficiency in deep sleep and REM sleep phases (Figure 4). Sleep efficiency was 57%. The arousal index was massively elevated at 90/h, of which 89/h were attributable to breathing and 1/h to periodic leg movements. The periodic movements of limbs (PML) index was normal at 0/h. The average heart rate was 75 beats per minute (Figure 4). The patient slept predominantly on her side. Very loud, regular, and intermittent snoring was audible. The overall apnea-hypopnea index (AHI) was 122 and 89 during REM sleep, the mean oxygen saturation was 91%, and the minimum oxygen saturation was 69%. Obstructive apneas, accompanied by significant drops in oxygen saturation, occurred throughout the recording, but particularly during REM sleep. The apneas and hypopneas lasted an average of 16 and 20 seconds, respectively, with maximum durations of 46 and 37 seconds. OSAS was diagnosed, and nocturnal APAP therapy (7/14 cmH₂O) was prescribed. The patient’s current medication consisted solely of L-thyroxine.

## Discussion

The index patient is of interest because she carries a novel variant in the COL1A2 gene, which phenotypically manifested as joint and skin hyperlaxity, brachydactyly present since childhood, multiple nevi, ptosis, coxalgia, hair loss, and presumably OSAS. While joint and skin hyperlaxity [7], nevi [8], ptosis [9], and hair loss [10] have previously been described in association with mutations in the COL1A2 gene (Table 1), OSAS has not yet been reported as a phenotypic feature of a COL1A2 variant. However, OSAS has been described as a clinical feature in various other CTDs [6,11]. For example, OSAS has been described in patients with Marfan syndrome, hypermobility spectrum disorders, osteogenesis imperfecta, polymyositis, systemic lupus erythematosus [12], EDS [13], and systemic sclerosis [14]. Some studies have found that OSAS occurs in up to 60% of patients with CTDs [15]. It is believed that OSAS in CTDs is likely caused by chronic inflammation, medication side effects, and structural changes. The increased risk of OSAS in CTDs is also attributed to anatomical abnormalities, such as increased tissue compliance (flexibility) of the upper airways, a narrow palate, or structural skeletal problems that allow the airways to collapse during sleep.

**Table 1:** Comparison of phenotypic features between the index patient and previously reported cases Whether OSAS, hair loss, hypotelorism, and umbilical hernia were actually attributable to the COL1A2 variant remains questionable. OSAS: obstructive sleep apnea syndrome

Phenotype	Previously reported	Index patient
Hyperlaxity of joints	yes [7]	yes
Sleep apnea syndrome	no	yes
Hair loss	no	yes
Short stature	yes [6]	yes
Hypoacusis	yes [6]	yes
Ptosis	yes [9]	yes
Nevi	yes [8]	yes
Hypotelorism	no	yes
Umbilical hernia	no	yes
Brachydactyly	yes [16]	yes
Foot deformity	yes [17]	yes
Facial dysmorphism	yes [18]	no

The variant c.980_984dup (p.Ile329Alafs*72) in COL1A2 was suspected to be responsible for OSAS in the index patient because it resulted in an amino acid change, it was predicted to be pathogenic, it occurred in the heterozygous form, the patient had developed features of CTD, such as hyperlaxity of skin and joints, hair loss, hypoacusis, ptosis, and nevi, her son had also developed OSAS, and OSAS is a common manifestation of CTD. The mutation likely resulted in a loss of the protein, as loss of function is a known pathophysiologic mechanism for COL1A2-associated diseases. The variant is not listed in the gnomAD population database.

The pathophysiology underlying the development of OSAS in COL1A2-associated CTDs is not readily explained. However, it can be surmised that the phenotypic abnormalities of the COL1A2 variants are related to the function of the COL1A2 protein in the formation of type I collagen, which is abundant in cartilage, bone, tendons, skin, and the sclera [19]. The COL1A2 protein is involved in the assembly and processing of procollagen molecules into mature collagen fibers, which are crucial for the strength and stability of the tissue. The COL1A2 protein is also involved in wound healing and elastin production, cell proliferation, cell migration, and elastin synthesis [8]. In EDS, the occurrence of OSAS and sleep-related breathing disorders can be explained by connective tissue laxity [20]. It is also suspected that damage to the temporomandibular joint or the cervical spine is present, which presumably contributes to airway obstruction in OSAS [20].

Limitations of the study included the fact that no functional tests were performed to confirm the pathogenicity of the variant, that the mutation was not confirmed by Sanger sequencing, the index patient’s son did not undergo genetic testing, no segregation analysis was performed, and a comprehensive family history could not be obtained due to complex family conflicts that led to communication barriers. Another limitation is that APAP therapy was only partially effective for insomnia, suggesting that additional causative factors were involved.

## Conclusions

This case raises the concern that OSAS may be hereditary due to a variant in the COL1A2 gene, that it may represent the dominant phenotypic trait of a COL1A2 mutation, and that the inheritance of OSAS may follow an autosomal dominant pattern, with OSAS and CTD appearing in successive generations. However, further research is needed to confirm this possibility. In patients with a positive family history of OSAS who also exhibit typical features of CTD, hereditary OSAS should be suspected, and genetic testing for variants in genes associated with CTD should be performed. Coexisting OSAS in CTD patients can increase cardiovascular risk and impair overall quality of life.
